# Cohort Profile Update: The Young Lives study

**DOI:** 10.1093/ije/dyag087

**Published:** 2026-06-16

**Authors:** Marta Favara, Maria De Los Ángeles Molina, Alan Sánchez, Kauser Mohsini, Tassew Woldehanna, Alula Pankhurst, Ellanki Revathi, Antonio Campos, Katherine Curi-Quinto, Vanessa Rojas

**Affiliations:** Oxford Department of International Development, University of Oxford, Oxford, United Kingdom; Oxford Department of International Development, University of Oxford, Oxford, United Kingdom; Oxford Department of International Development, University of Oxford, Oxford, United Kingdom; Grupo de Análisis para el Desarrollo (GRADE), Lima, Peru; Oxford Department of International Development, University of Oxford, Oxford, United Kingdom; Department of Economics, Addis Ababa University, Addis Ababa, Ethiopia; Pankhurst Development Research and Consulting, Addis Ababa, Ethiopia; Centre for Economic and Social Studies (CESS), Hyderabad, India; Grupo de Análisis para el Desarrollo (GRADE), Lima, Peru; Instituto de Investigación Nutricional (IIN), Lima, Peru; Grupo de Análisis para el Desarrollo (GRADE), Lima, Peru

**Keywords:** longitudinal cohort, birth cohort, life course, childhood poverty, transition to adulthood, low- and middle-income countries, mental health, Ethiopia, India, Peru

Key Features

- Young Lives is a longitudinal study that was set up in 2001, tracing a Younger Cohort born in 2000–1 and an Older Cohort born in 1994–5 through childhood, adolescence, and adulthood in four low- and middle-income countries: India, Ethiopia, Peru, and Vietnam.
- More than two decades of follow-up provide opportunities to investigate the long-term effects of growing up in poverty on cognitive skills development, educational attainment, employment opportunities, and physical and mental health.
- Five in-person survey rounds were completed by 2016. Round 6 was carried out over five phone calls during the COVID-19 pandemic. The latest in-person survey, Round 7, was conducted in 2023–4 in India, Ethiopia, and Peru, interviewing 7123 participants from the Younger Cohort and Older Cohort (81% of the original sample), aged 22 and 29 years, respectively.
- Round 7 incorporates new measures of mental health, questions on exposure to violence, risky behaviours, sexual and reproductive health, foundational cognitive skills, and job attributes that young people value. Additionally, hair samples were collected to measure cortisol. Furthermore, qualitative data collection took place in Ethiopia and Peru, with particular emphasis on young people’s health and well-being.
- Data from Round 7 of the Young Lives study are publicly available through the UK Data Service (https://datacatalogue.ukdataservice.ac.uk/series/series/2000060#access-data).

## The original cohort

The Young Lives study is a multi-country, mixed-methods, longitudinal study that was established in 2001 with the aim of investigating the causes and consequences of growing up in poverty in the developing world and to inform policy to improve the lives of disadvantaged children and young people [1].

Since 2002, Young Lives has followed the lives of ∼12 000 children—now young adults—born into poverty in Ethiopia, India (states of Andhra Pradesh and Telangana), Peru, and Vietnam. In each country, the baseline sample comprises two cohorts: the Younger Cohort of 8064 children born in 2000–1 and the Older Cohort of 3722 children born in 1994–5. More details about the original sampling design are available on our website.

Additionally, Young Lives has followed a subset of 200 participants as part of a qualitative study since 2007 (five rounds in Peru, six in Ethiopia, and four in India and Vietnam), which includes semi-structured interviews and participatory data collection with children, their families, and key informants from their communities [2].

The original Young Lives cohort profile was published in 2012. It describes the study participants, the data collected in Rounds 1–3 of both the quantitative and qualitative surveys, and the attrition rates [1]. An update was published in 2021. It describes Rounds 4 and 5 of the quantitative surveys and the data collected through three phone calls (Calls 1–3) in 2020, as part of Round 6 [3]. This paper describes the latest survey, Round 7, conducted in 2023 to capture participants’ transition into adulthood. Round 7 was administered through in-person interviews, except within two sites of the Amhara region of Ethiopia, where phone interviews were conducted due to armed conflict.

## What is the reason for the new data collection?

From its initial focus on childhood poverty that characterized the first five survey rounds, the Young Lives study has shifted focus in Rounds 6 and 7 as participants transition into adulthood, to investigate the factors that promote and constrain the equality of opportunity and social mobility across the first three decades of life and between generations. Round 6 was carried out in 2020–1 during the COVID-19 pandemic and included five phone surveys: three calls conducted in 2020 [3] and two additional calls completed in 2021. The attrition rate of the 2020 calls is reported in Favara *et al.* (2021) [3] and the final Round 6 attrition rate is reported in Molina *et al.* (2025) [4].

In 2023, 7 years after the last face-to-face interview, a seventh round of in-person data collection was conducted in Ethiopia, India, and Peru. Unfortunately, Round 7 was not conducted in Vietnam due to changes in governmental procedures on the international transfer of personal data [5].

## What will be the new areas of research?

Round 7 incorporates survey modules from previous rounds to enable cross-cohort comparisons and life-course analysis, and includes additional modules to reflect both the evolving life stages of Young Lives participants and significant disruptions such as the COVID-19 pandemic, armed conflict, and social and economic crises:

Education and skills: reading comprehension and foundational cognitive skills tests were administered to the Younger Cohort to trace the learning progression from adolescence to adulthood.

Employment: willingness-to-pay questions designed to understand the job attributes that young people value, to facilitate research on labour supply. Furthermore, an enhanced employment module was included to more precisely understand their employment situation. This is important in the context of widening labour-market inequalities and increased job insecurity, which have been exacerbated by the COVID-19 pandemic (especially in Peru and India), armed conflict in Ethiopia, increased internal and international migration, and climatic shocks.

Health and well-being: expanded mental health scales to assess the impact of multiple, intersecting crises on the well-being of our participants now that they are adults. The collection of hair samples to analyse cortisol levels will reveal how early age and recent stressful experiences impact mental and physical health, alongside other outcomes in adulthood.

Family lives: questions on exposure to violence, risky behaviours, and sexual health, to understand young people’s experiences and behaviours. Our research will also examine how global crises affect household gender dynamics, fertility, and family formation decisions.

## Who is in the cohort?

**Figure 1:**
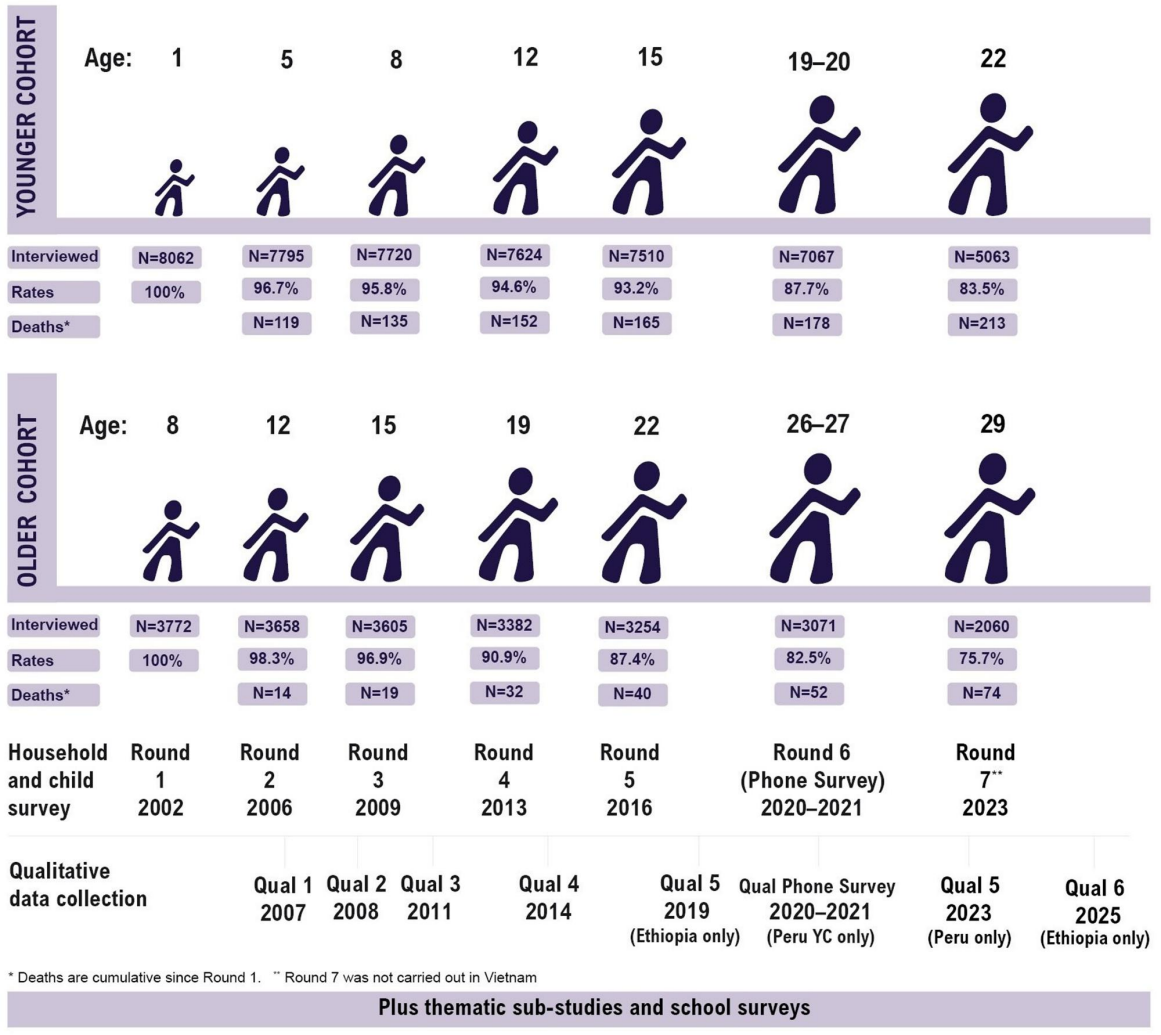
Young Lives survey rounds with cohorts’ ages, sample sizes, and response rates.

Round 7 was conducted between mid-2023 and early-2024 when participants were approximately aged 22 years (Younger Cohort) and 29 years (Older Cohort). Of the original 2002 sample, counting 8784 participants in Ethiopia, India, and Peru, 7123 were surveyed in Round 7 (81.0% total retention rate). Table 1 describes the Round 7 sample according to the baseline characteristics measured in Round 1. Follow-up rates were highest in India and lowest in Ethiopia (Fig. 1).

**Table 1:** Follow-up rates for Round 7 sample according to baseline characteristics.

	Ethiopia				India				Peru			
	Round 1	Round 7			Round 1	Round 7			Round 1	Round 7		
	2002	2023	%	*P* value	2002	2023	%	*P* value	2002	2023	%	*P* value
Total	2999	2231	74.39		3019	2673	88.54		2766	2219	80.22	
Cohort	2999				3019				2766			
Older Cohort	1000	696	69.60	.000	1008	847	84.03	.000	714	517	72.41	.000
Younger Cohort	1999	1535	76.79		2011	1826	90.80		2052	1702	82.94	
Sex	2999				3019				2766			
Male	1559	1162	74.53	.851	1572	1397	88.87	.555	1353	1123	83.00	.0003
Female	1440	1069	74.24		1447	1276	88.18		1413	1096	77.57	
Area of residence	2999				3019				2766			
Rural	1948	1525	78.29	.000	2260	2080	92.04	.000	830	657	79.16	.3562
Urban	1051	706	67.17		759	593	78.13		1936	1562	80.68	
Caregiver’s education	2756				3017				2764			
Incomplete primary	2174	1629	74.93	.203	1920	1735	90.36	.000	835	655	78.44	.1174
At least complete primary	582	421	72.34		1097	936	85.32		1929	1563	81.03	
Wealth tertile	2974				3014				2755			
Lowest wealth tertile	1004	773	76.99	.000	1012	931	92.00	.000	924	744	80.52	.53
Middle wealth tertile	980	753	76.84		998	904	90.58		913	723	79.19	
Top wealth tertile	990	686	69.29		1004	833	82.97		918	744	81.05	

## What has been measured?

### Education and skills

We readministered the reading comprehension test to the Younger Cohort, to trace the development of reading comprehension skills from age 12 years to age 22 years (Table 2). Furthermore, we administered part of the Rapid Assessment of Cognitive and Emotional Regulation (RACER), completed by the Younger Cohort in Ethiopia and Peru in Round 5, to measure changes in inhibitory control and working memory between ages 12 and 22 years [6].

**Table 2:** Information collected in Young Lives Round 7 by research stream.

Research stream	Information collected
Education and skills	Education history, current education, school/college characteristics, socio-emotional skills, foundational cognitive skills, digital skills
Health and well-being	Household food security, dietary diversity, anthropometry, physical health, household access to health services, subjective well-being, mental health, hair samples for cortisol analysis to investigate stress, risky behaviours, healthy behaviours, violence, sexual health, health at birth of next generation
Employment	Employment and job characteristics in week prior to the interview and in the past 12 months, job quality, work training and skills, work activities and household shocks, willingness to pay, impact of COVID-19 pandemic on work
Family lives	Household composition and demographic characteristics, household socio-economic status, household shocks and coping strategies, household transfers and access to public programmes, marital and living arrangements, fertility, exposure to violence, time use, movement history

### Health and well-being

For the first time in the Young Lives study, hair samples were collected for the analysis of cortisol levels, as a proxy for the preceding months of hypothalamic–pituitary–adrenal axis activity, which is commonly implicated in the aetiology of stress and depression [7]. Laboratory analysis is currently underway [8]. Additionally, we incorporated a more comprehensive set of mental health and subjective well-being measures through internationally validated instruments—the Perceived Stress Scale (PSS-10) and the International Trauma Questionnaire (ITQ-9), the latter in Ethiopia only [9]. The in-person interview also enabled us to collect anthropometric measures such as completed height for the Younger Cohort as adults, as they were only 15 years old during the last face-to-face interview.

### Employment

For the first time, we asked detailed information about the attributes of the participants’ main job during the 7 days prior to the interview (including earnings, working hours, contract, benefits, and social protection) in addition to information on their main occupation over the last 12 months. We included a survey module on willingness to pay for job characteristics with the aim of understanding the non-wage attributes that young people value in the workplace [10]. Participants were presented with four hypothetical jobs, each described by three of four randomized job characteristics—working hours, job flexibility, type of contract, and workplace gender composition—along with wages. They were then asked to assign a score to each job and estimate the probability of obtaining each job within 1 year.

### Family lives

We also made use of new technologies to administer a self-administered questionnaire (SAQ) in Peru and audio-computer assisted self-interviews (ACASIs) in Ethiopia and India, which involved participants interacting with a computer screen and either reading questions (SAQ) or hearing questions through headphone audio (ACASIs), the latter to facilitate the administration in low-literacy contexts. Using self-administrated tools enables us to ask sensitive questions on exposure to violence, risky behaviours, and sexual health [11]. In Ethiopia, in the context of the armed conflict in Tigray and Amhara, information on individual perceptions of security, violent and traumatic experiences, difficulties in access to services, education, employment, and healthcare due to conflict was collected through ACASIs, with less sensitive questions included as part of the main survey.

## What has it found? Key findings and publications

The preliminary findings from Round 7 are summarized below. Table 3 presents findings stratified by cohort and Table 4 presents findings stratified by sex.

**Table 3:** Selected characteristics of the study sample in Round 7, stratified by cohort.

	Younger Cohort (%)			Older Cohort (%)		
Research theme	Ethiopia	India	Peru	Ethiopia	India	Peru
**Education and skills**						
**Educational attainment**						
Currently enrolled in formal education	34.1	31.7	47.5	5.2	2.7	10.4
Completed upper-secondary education	26.3	66.4	91.4	20.6	56.9	86.6
Ever enrolled in post-secondary education	25.1	63.5	68.0	37.2	47.3	59.8
Completed post-secondary education	20.4	33.4	13.7	35.5	42.8	43.3
**Health and well-being**						
**Nutrition and food security**						
Underweight	22.9	24.0	2.0	17.6	11.6	1.0
Normal	72.6	55.0	56.2	68.8	49.5	32.4
Overweight or obese	4.5	21.0	41.8	13.6	38.9	66.6
At least mild food insecurity	74.1	80.2	59.1	74.5	76.4	54.7
**Mental health**						
Symptoms of at least mild anxiety	21.6	8.9	33.2	25.9	14.0	33.0
Symptoms of at least mild depression	15.6	9.8	29.8	18.7	10.7	21.2
Symptoms of at least moderate stress	61.8	59.0	71.6	63.1	59.1	65.7
Gender gap for at least mild anxiety	0.5	4.2	18.5	1.5	12.3	16.5
**Employment**						
**Labour market and job quality**						
Employed in week before survey	55.1	54.4	67.6	61.2	76.5	84.7
Gender gap in employment week before survey	29.5	33.6	18.3	38.1	36.4	21.2
Has a written contract	9.7	6.7	22.2	28.6	8.2	36.3
Worked in week before survey with written contract	9.5	6.5	21.6	27.5	8.1	35.7
**Education and employment**						
Studying only	18.2	16.2	11.3	2.1	0.8	1.0
Studying and working	14.2	13.1	33.7	2.6	1.7	9.2
Working only	51.4	49.8	49.1	69.7	78.5	82.2
Not in education, employment, or training (NEET)	16.2	20.9	5.8	25.5	19.0	7.5
**Family lives**						
**Family formation**						
Ever married/cohabited by Round 7	12.7	24.6	27.4	56.1	74.9	66.5
Gender gap for marriage/cohabitation before legal age	4.2	9.4	7.2	12.5	18.3	14.2
Being parent	9.4	16.1	22.0	47.4	59.9	56.3
Gender gap for parenthood before age 19 years, %	6.4	17.0	14.8	12.9	26.0	22.1
Experienced any form of intimate-partner violence	18.4	33.3	28.1	21.9	24.3	33.1
**Risky behaviours**						
Ever smoked	7.8	10.0	47.0	13.3	9.9	11.3
Ever drank heavily	9.3	3.8	49.8	14.7	4.3	61.4
Ever consumed any illegal drugs	5.1	2.0	14.3	8.9	1.5	16.1

**Table 4:** Selected characteristics of the study sample in Round 7, stratified by sex.

	Women (%)			Men (%)		
Research theme	Ethiopia	India	Peru	Ethiopia	India	Peru
**Education and skills**						
**Educational attainment**						
Currently enrolled in formal education	28.4	19.2	42.1	21.9	25.5	35.6
Completed upper-secondary education	22.7	49.3	78.6	18.9	50.7	81.9
Ever enrolled in post-secondary education	33.9	51.9	68.7	24.4	64.3	63.4
Completed post-secondary education	30.2	33.0	22.7	20.4	39.5	18.5
**Health and well-being**						
**Nutrition and food security**						
Underweight	23.4	24.5	2.4	19.5	16.3	1.5
Normal	68.2	51.0	53.1	74.3	55.2	59.4
Overweight or obese	8.4	24.6	44.6	6.1	28.6	39.0
At least mild food insecurity	74.6	75.6	62.4	73.9	82.1	53.6
**Mental health**						
Symptoms of at least mild anxiety	22.9	14.2	42.1	22.9	7.1	24.1
Symptoms of at least mild depression	17.9	12.3	35.2	15.4	8.0	20.3
Symptoms of at least moderate stress	64.1	57.8	78.2	60.4	60.1	62.2
**Employment**						
**Labour market and job quality**						
Employed in week before survey	41.2	44.0	61.9	73.4	77.4	81.4
Has a written contract	22.9	8.0	25.3	12.8	7.0	26.9
Worked in week before survey with written contract	21.7	7.9	24.5	12.6	6.8	26.2
**Education and employment**						
Studying only	18.4	13.3	9.9	8.5	9.4	7.9
Studying and working	8.6	3.7	30.0	12.3	14.9	26.0
Working only	43.0	46.9	48.9	70.1	69.9	65.0
Not in education, employment, or training (NEET)	30.0	36.1	11.1	9.0	5.8	1.1
**Family lives**						
**Family formation**						
Ever married/cohabited by Round 7	36.8	61.9	45.3	16.5	20.8	27.5
Marriage/cohabitation before legal age	7.3	16.8	11.1	0.5	4.2	2.4
Being parent	32.3	47.9	39.8	11.0	13.6	20.0
Parenthood before age 19 years	10.6	21.0	23.8	2.2	0.9	7.5
Experienced any form of intimate-partner violence	22.9	32.0	34.6	25.9	35.3	23.9
**Risky behaviours**						
Ever smoked	6.0	3.5	31.6	12.7	15.9	63.6
Ever drank heavily	4.0	0.5	41.2	17.2	7.3	64.0
Ever consumed any illegal drugs	2.7	2.1	8.9	9.5	1.5	20.7

### Education and skills

An increasing number of young people are completing upper-secondary education. In India, upper-secondary completion had improved over the last 7 years; at age 22 years, 66.4% of the Younger Cohort had completed upper secondary (Grade 12) compared with 56.9% of the Older Cohort at age 22 years [12]. In Peru, 91.4% of the Younger Cohort had completed upper secondary (Grade 11) by 2023 compared with 86.6% of the Older Cohort in 2016 [13]. In Ethiopia, around a quarter of the Younger Cohort had completed upper secondary (Grade 12) by age 22 years; university enrolment declined due to armed conflict and a change in the university entrance exam [14]. In Peru, Younger Cohort participants who scored highest in reading comprehension tests were more likely to be enrolled in or to have completed university; improvement in reading comprehension skills between ages 15 and 22 years corresponded to educational attainment during the same period [13]. By contrast, a stagnation was observed in India; more schooling years and higher secondary-school completion rates overall did not translate into an improvement in reading comprehension skills between ages 15 and 22 years [12]. Similarly, in Ethiopia, the reading comprehension test performance did not improve between ages 15 and 22 years for the Younger Cohort, even for those who had completed upper secondary and were enrolled in tertiary education [14].

### Health and well-being

In India and Peru, mental health indicators have worsened for women since the COVID-19 pandemic compared with men, consistently with global trends. Indicators of mental health and subjective well-being tend to be lower among socio-economically disadvantaged groups in Ethiopia and India; the opposite is true in Peru [9]. All three countries show high rates of at least mild food insecurity, similarly to global trends; most households are moderately or severely food-insecure [15]. Early-life inequalities are associated with high food insecurity, but they could also be partially explained by economic and food system crises after COVID-19. In Peru, the prevalence of those overweight or obese is high and has increased over time [16].

### Employment

There is a persistent gender gap in the distribution of work; women are less likely to be employed than men and spend more time on unpaid care work that relies upon and reinforces gender-based inequalities (Table 4) [17–19]. In Peru, the gender gap in employment has widened for the Younger Cohort since the COVID-19 pandemic [17]; in India, it has remained persistently high but unchanged [19]. The prevalence of those not in education, employment, or training (NEET) has increased in the Younger Cohort at age 22 years compared with the Older Cohort at age 22 years, particularly in Ethiopia [18]. In both Ethiopia and Peru, a higher proportion of the Older Cohort are classified as NEET compared with the Younger Cohort. Overall, women are much more likely to be classified as NEET than men, with the absolute difference ranging from 10.0% in Peru to 21.0% in Ethiopia and 30.3% in India.

### Family lives

Across all three countries, women are more likely to experience early marriage and early parenthood than men, although the proportion of women who are married, cohabiting, or are parents before the legal age or by age 22 years has decreased in the Younger Cohort compared with the Older Cohort (Table 4) [17–19]. Young people from poorer, disadvantaged groups are more likely to experience early marriage and early parenthood. In Ethiopia, participants in conflict-affected regions of Tigray and Amhara reported higher exposure to shocks, such as the disruption of public services and utilities, interruption in access to the communication infrastructure, worse mental health, nutrition, and food security, and increasingly precarious labour and education conditions [14, 15, 18, 20]. This has caused a decline in upper-secondary completion and enrolment in tertiary education [14].

The emerging findings from >20 years of the study are summarized in our thematic legacy reports on education and skills, health and well-being, employment, and family lives, released in March 2026 (https://www.younglives.org.uk/publications). Notable findings since the publication of the last Cohort Profile Update include:

Education and skills: early-life exposure to rainfall shocks in Peru impedes the development of foundational cognitive skills, particularly during the *in utero* period, and negatively affects household food security and early nutritional status [21]. Public works programmes and social protection schemes can attenuate the adverse effects of early-childhood poverty and negative shocks on the development of foundational cognitive skills [22, 23]. Foundational cognitive skills at age 12 years predict educational attainment (secondary-school completion and university enrolment) through adolescence and into young adulthood [24].

Health and well-being: food insecurity increased during the COVID-19 pandemic [25], with food-insecure households reporting higher rates of symptoms consistent with depression and anxiety across all four Young Lives countries [26]. In Ethiopia, the outbreak of violent armed conflict in November 2020 worsened mental health problems. The rates of anxiety were three times higher than those pre-conflict. The rates of depression increased substantially from 16% to 25%; males reported greater increases in anxiety and females reported greater increases in depression [27]. Round 5 of the qualitative study in Peru, conducted in 2023, revealed that mental health problems were exacerbated by the COVID-19 pandemic and lockdown, particularly for women, who experienced higher levels of stress and anxiety, compounded by additional unpaid care work and domestic responsibilities, an increase in gender-based violence, and the loss of educational and employment opportunities [28, 29]. Younger participants tended to report greater emotional distress and uncertainty during and after the pandemic, whereas older participants often highlighted coping strategies linked to family support, employment stability, or personal goals. Furthermore, social stigma, lack of social protection, and barriers to accessible and high-quality mental health services deter young people from seeking help or treatment [9].

Employment: in Vietnam, job loss owing to the COVID-19 pandemic contributed to a 5.9% rise in the probability of experiencing symptoms of mild or severe anxiety [30]. By the end of 2020, the COVID-19 pandemic had contributed to a widening of the gender employment gap by 18.0% in India, 17.5% in Peru, and 9.5% Vietnam. The unequal distribution of care work within households explained between 15.8% (Vietnam) and 21.1% (Peru) of this change [31].

## What are the main strengths and weaknesses?

### Strengths

The main strengths of the Young Lives study are related to the multinational sample and diversity of the populations included, which enhance the external validity of our findings to other contexts in the global south. The mixed-methods design, featuring qualitative, semi-structured interviews with a subset of participants in addition to the main survey, ensures a comprehensive, holistic approach to investigating the long-term effects of childhood poverty and supporting policy recommendations. This is evidenced by lived-experience accounts from Round 5 of the qualitative study in Peru, which complemented findings from the main survey to reveal how persisting gender and socio-economic inequalities were amplified by the COVID-19 pandemic [29]. Furthermore, the dual cohort design enables comparisons between two groups of participants born 7 years apart and hence comparisons of outcomes across years.

The Young Lives study has had low attrition rates over >20 years of data collection, with 81% of the original sample participating in Round 7. Another strength is the ability to adapt to the context and circumstances, enhancing the quality and cultural appropriateness of the data collected. For example, in Round 7, participants were offered different language options and questionnaires were translated into local languages. In Ethiopia, the survey could be conducted either in Amharic, Oromo, or Tigrinya, in English or Telugu in India, and in Spanish in Peru. Furthermore, ACASIs proved to be an effective tool for collecting sensitive information on participants’ exposure to violence by assuring privacy and confidentiality [11, 32]. The ACASIs were recorded in local languages with male/female voices matched to the sex of the participant [11].

Finally, Young Lives offers opportunities for data linkages, enabling research into how compounding crises shape the life course. We recently launched the new Young Lives Research Hub on Climate Change and Environmental Shocks, with the aim of delivering new research on how life-course exposure to climate shocks impact young people’s development and well-being in the global south (https://www.younglives.org.uk/news/global-climate-summit).

### Weaknesses

The Young Lives study is not nationally representative, although it captures ethnically and linguistically diverse populations. The study does not follow participants who move across national borders, presenting a disadvantage when investigating migration, which tends to increase at our participants’ current life stage [4]. While the overall attrition rate between Rounds 1 and 7 is relatively low compared with those of other cohort studies, during Round 7, we observed a larger attrition rate in Ethiopia due to the ongoing armed conflict in Tigray and Amhara, and less severe conflict across all regions. However, we minimized conflict-related attrition by administering a phone survey, featuring a shortened form of the Round 7 survey instrument, in which 8.7% of observed participants from Ethiopia took part. Nonetheless, the attrition rate in Ethiopia is still larger than those in other countries. Additionally, due to changes in international data-transfer laws in Vietnam, we were unable to conduct Round 7 there.

## Can I get hold of the data? Where can I find out more?

Data from Round 7 of the Young Lives study are publicly available through the UK Data Service (https://datacatalogue.ukdataservice.ac.uk/series/series/2000060#access-data) (4 May 2026, date last accessed). For further information, the Young Lives study team can be contacted at younglives@qeh.ox.ac.uk.

## Ethics approval

Ethics approvals for the Young Lives study were obtained by the Social Sciences and Humanities Inter-Divisional Research Ethics Committee (SSH IDREC) at the University of Oxford: in October 2022 (Ref.: SSH/ODID_C1A_22_088); in April 2023 (Ref.: R85604/RE001); in February 2024 (Ref.: C1A_23_002). The study was also approved in each study country: in Ethiopia, approval was granted by the National Research Ethics Review Board of the Ethiopian Ministry of Education (Ref.:17/152/702/23) and the College of Education and Behavioural Studies Institutional Review Committee, Addis Ababa University (Ref.: CEBS_IRC/009/2023); in India by the Centre for Economic and Social Studies in Hyderabad and the Institutional Ethics Committee for Biomedical Research at the Institute of Genetics & Hospital for Genetics Diseases (Ref.:766/1/IG/IECBR/2023); and in Peru by the Instituto de Investigación Nutricional (Ref.:N°053–2023/CIEI-IIN). Study participants were asked for their informed consent to participate in the study and additional consent was required for additional survey components (reading comprehension test, self-administered questionnaire, hair samples, computerized cognitive tasks, and anthropometrics). All respondents received a consultation guide that included information about physical and mental health, and information and contact details of local public resources available to provide support if needed. Additionally, they received small economic compensation for their time, considering that the opportunity cost for our adult participants is higher now that they are of working age: ETB 300 (4.5 GBP) in Ethiopia; INR 1250 (10 GBP) in India; and PEN 50 (12 GBP) in Peru.

## Data Availability

The data underlying this article are available in the UK Data Service, at http://doi.org/10.5255/UKDA-SN-9538-1.
